# Technical aspects of gated SPECT MPI assessment of left ventricular dyssynchrony used in the VISION-CRT study

**DOI:** 10.1007/s12350-020-02122-3

**Published:** 2020-05-11

**Authors:** Amelia Jimenez-Heffernan, Sadaf Butt, Claudio T. Mesquita, Teresa Massardo, Amalia Peix, Alka Kumar, Chetan Patel, Erick Alexanderson, Luz M. Pabon, Ganesan Karthikeyan, Claudia Gutierrez, Victor Marin, Ernest Garcia, Diana Paez

**Affiliations:** 1 Hospital Universitario Juan Ramon Jimenez, Ronda Exterior Norte s/n, 21005 Huelva, Spain; 2 Oncology and Radiotherapy Institute (NORI), Islamabad, Pakistan; 3 Hospital Universitario Antonio Pedro, Niteroi, Brazil; 4 Hospital Clinico Universidad de Chile, Santiago, Chile; 5 Nuclear Medicine Department, Institute of Cardiology, La Habana, Cuba; 6 Dr. B L Kapur Memorial Hospital, New Delhi, India; 7 All India Institute of Medical Sciences, New Delhi, India; 8 Instituto Nacional de Cardiologia Ignacio Chavez, Mexico, DF Mexico; 9 Fundacion Valle del Lili, Cali, Colombia; 10 Fundacion Cardioinfantil, Bogota, Colombia; 11 Emory University, Atlanta, GA USA; 12 Nuclear Medicine and Diagnostic Imaging Section, International Atomic Energy Agency, Vienna, Austria

**Keywords:** MPI, Dyssynchrony, Gated SPECT, Heart failure, Resynchronization therapy

## Introduction

Clinical interest continues in identifying heart failure patients who will improve with cardiac resynchronization therapy (CRT). Since the advent of applying phase analysis to measure the synchronicity of the regional LV onset of mechanical contraction^1^ and the availability, automation and reproducibility of measuring phase from gated SPECT MPI (gMPI) associated with this process there has been a need to properly evaluate this methodology. This need prompted the International Atomic Energy Agency (IAEA) to fund a multicenter trial to study this opportunity. The trial was titled ‘‘Value of intraventricular synchronism assessment by gated SPECT myocardial perfusion imaging in the management of heart failure patients submitted to cardiac resynchronization therapy’’ (IAEA VISION-CRT). Several reports on the findings of this trial are already published^2^–^4^ and other reports are forthcoming.

The purpose of this report is to describe the technical considerations undertaken in this trial to assure valid results and the algorithms in ECTb4 used to measure synchronicity-pertinent LV parameters. Some of the descriptions below are specific to using ECTb4. The quality control, the processing technical tips and algorithms descriptions are universal to technologists, physicians and industries interested in assessing LV mechanical dyssynchrony (LVdys).

## Technical Aspects of gMPI Acquisition and Reconstruction

### Acquisition

The SPECT scans are performed approximately 30 minutes post rest injection using 740-1110 MBq (20-30 mCi) of 99mTc-sestamibi or tetrofosmin. SPECT images are usually acquired on dual-headed cameras with high-resolution low energy collimators, using 180° orbits and a standard resting protocol.

Gating errors can cause errors in the measurement of LV dyssynchrony,^5^ usually seen in patients with atrial fibrillation or ectopic beats. These may result in a reduction of counts in the late frames and are typically detected as flickering in gated short-axis (SA) and/or gated planar projection cine displays; the drop in counts has the effect of making the LVdys measurement appear more synchronous than in reality. Patients being imaged to guide CRT are required to be in sinus rhythm. Those cases experiencing arrhythmias are instrumented with conventional pacemakers to bring them to sinus rhythm before being considered for enhancement to biventricular pacing with CRT, which helps but does not totally eliminate the gating error. Because of this concern, a 100% (50%-150%) R-to-R gating window is used. Another approach is to use a narrower R-to-R window such as 40% (80%-120%) R-to-R. This approach works best in systems where the acquisition per projection can be constrained to a preset number of myocardial counts per projection, otherwise projections with more rejected beats would have less counts and can create reconstruction artifacts.

**Figure 1:**
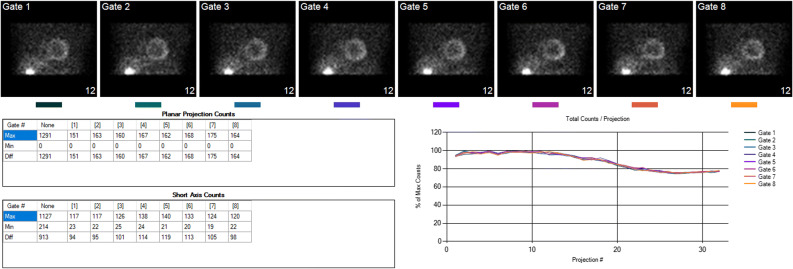
Quality control page in ECTb4 program provides information regarding gating and count statistics. Top images are the gated planar projection from an eight-frame acquisition. Gating errors would cause at least the image in gate 8 to flicker during rotation cine-display. The curves represent % maximal counts per planar projection and are color-coded to each gated frame. Since all curves are superimposed, it means that counts were not dropped at the end of the R-R cycle and thus accurate gating.^11^ The two tables display the counts for each of the eight frames in the planar projections (top table) and the short-axis counts (lower table). Comparison of the maximum counts in the SA slices to Table 1 provide the technical quality of the acquisition

Either 8 or 16 frames per cardiac cycle are used for ECG gating. Since the measurement of phase is count sensitive, i.e., the more counts the more accurate the dyssynchrony measurement, the choice of number of frames is dictated by the number of counts per pixel in the gated LV myocardium (see Table 1). Thus, if all variables are the same except 8 or 16 frames, the myocardial pixels from the 8 frame gated study will have twice the counts as the 16 frames study, and although counter-intuitive will yield a more accurate determination of LV dyssynchrony. Temporal resolution of phase analysis is related to count density per myocardial pixel and has been shown to be 5.6° corresponding to 1/64 of the cardiac cycle in common clinical acquisitions using gated SPECT with 8 or 16 frames per cardiac cycle. Both the accuracy of the ECG gating as well as the appropriateness of the count density are monitored using specialized QC algorithms shown in Figure 1.

**Table 1:** Technical quality recommended for the accurate measurement of LV dyssynchrony from SPECT gMPI studies

Technical Quality	Max Counts in Gated LV
Adequate	> 80 (300 typical)
Marginal	20–80
Inadequate	< 20

### Reconstruction and Filtering

All images are reconstructed in the standard way using OSEM with 3 iterations and 10 subsets and filtered by a Butterworth filter, power 10, using a cut-off frequency of 0.3 cycles/mm. Although there are reports that the reconstruction approach does not have a strong impact on the LVdys calculation the goal is to optimizing the number of counts per pixel. Reconstructed trans-axial images are reoriented into SA slices used conventional methods.

Additional spatial-temporal filtering is performed on the reconstructed gated SA slices to reduce count noise. A 3 × 3 × 3 121/242/121 3D filter is first applied. A second adaptive filter is applied when a pixel located in the myocardium changes ≤ 5 counts between systole and diastole. This triggers that the counts in the pixel be replaced by the weighted average of its 8 2D neighbors.

### gMPI Processing and Quantification

The relatively linear relationship between myocardial thickening and myocardial count density in all segments is the basis for the assessment of LV dyssynchrony by phase analysis. Thickening is measured from the change in LV myocardial pixel counts from systole to diastole. Therefore, the time-activity curve of a myocardial segment is equivalent to its temporal thickening curve. A first-harmonic Fourier transformation is used to replace the discrete counts from each time-activity curve by a continuous thickening curve with good temporal resolution. The onset of myocardial contraction is then obtained from the crossing of the thickening curve with a line representing the average thickening for the curve.^1^,^6^ Both the measurements of thickening and onset of contraction measurements have been shown to be robust even in areas of hypoperfused myocardium allowing a good differentiation of patients with cardiac dysfunction from normal ones.^6^

### Processing Quality Control

Careful processing of gated SPECT MPI for dyssynchrony assessment is required to avoid and guarantee consistency. Therefore, a systematic approach is needed for optimal phase analysis.^7^ Automatic determination of gated center, radial extent and apex is robust, repeatable, and consistent with conventional processing. As always, operators are required to confirm that the algorithm correctly detected these landmarks and correct them manually if needed.

It is the base definition, the valve plane that carries the main error in both automatic and manual parameters creation.^7^ Automatic software may not be optimized for locating the gated valve plane for the precision required for phase analysis and thus the user must make sure processing parameters are appropriate.

**Figure 2:**
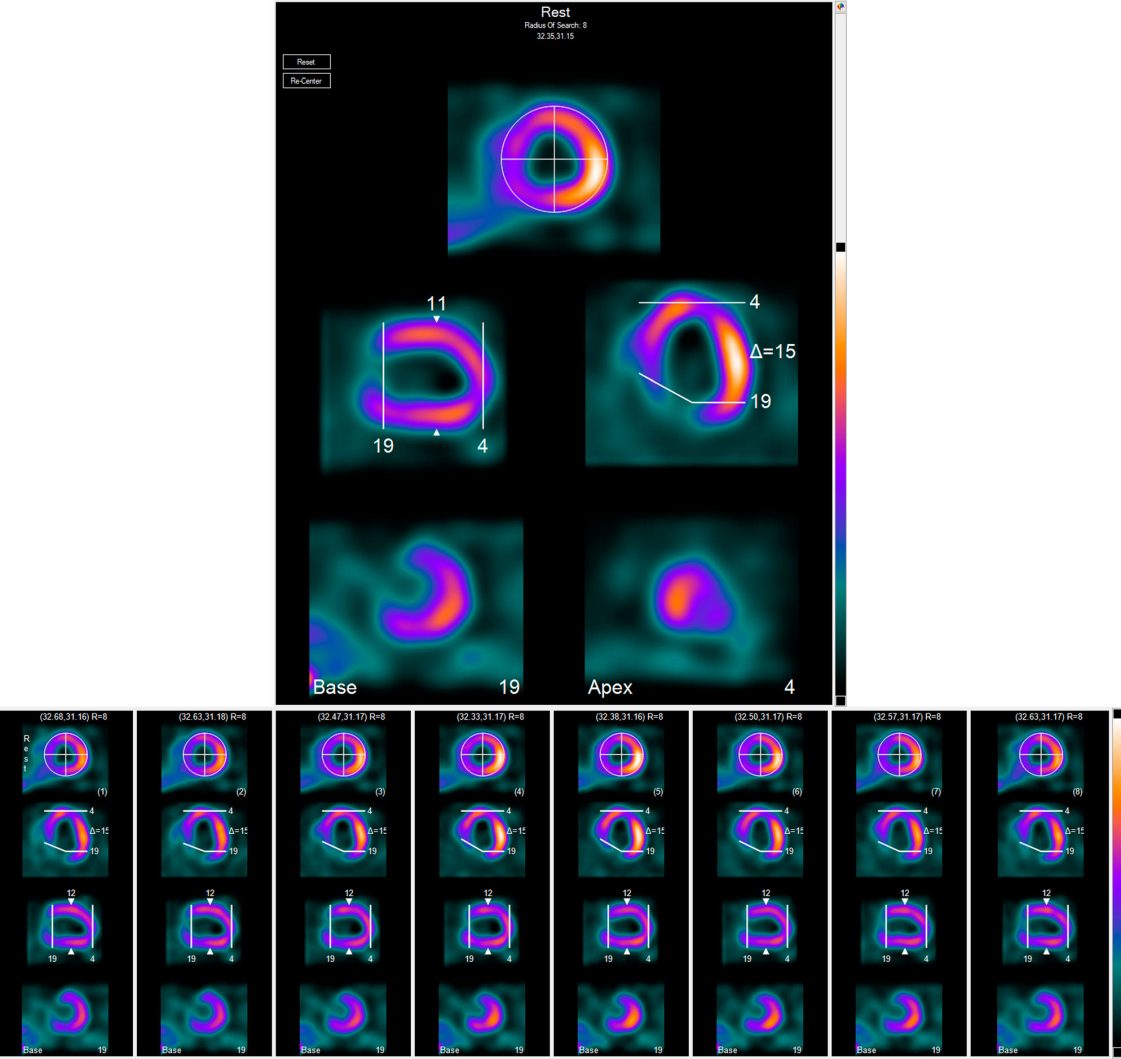
Correct base and apex determination to avoid phase artifacts. Top panel shows the automatic selection of base and apex from the ungated MPI images. Note the backward C representation of the correct SA base selection at 19. Also note the cap without a hole representation of the correct SA apical selection at 4. Lower panel shows how these selections from the ungated studies are replicated in the gated end-systolic slices (frame 4). Since this patient’s LVEF was 12% (i.e., < 35%) the algorithm does not move the base selection from systole to diastole. In studies where the algorithm fails to detect the base as specified or when the selection creates artifacts in the phase polar map at the LV base manual selection may need to be performed to remove the artifact and assure accurate results

When processing with ECTb, a correctly automatically detected LV base SA slice should look like a backwards C and the apex should look like a cap without a hole in it (Figure 2). This is true for both the ungated as well as the gated SA slices. This algorithm has been enhanced to emphasize accuracy in measuring LV dyssynchrony algorithm by starting with the base and apex detection from the ungated images as a starting point for iterative processing of gated parameters. Next, the base and apex selection from the ungated images are used in the ES gated image. In the subsequent gated images from ES to ED the base selection is moved outward depending on the patient’s initial LVEF as follows:

1. if the LVEF > 50% the base is allowed to move 2 slices in from ES to ED;
2. if the LVEF > 35% and ≤ 50%, the base is allowed to move 1 slice from ES to ED;
3. if the LVEF ≤ 35%, the base selection is not allowed to move.

These base selections can then be used to recalculate LVEF and repeat the base selection process until the described below are reduced or eliminated.

This base selection is designed to reduce created errors by including pixels from non-contracting regions that produce false areas of dyssynchrony in the phase map, usually at the base, and for the abnormal study it can change the location and severity of existing dyssynchrony. Moreover, techniques that increase the accuracy of phase analysis also promote the accuracy of all functional parameters.

## Quantification to Help Guide and Evaluate CRT

**Figure 3:**
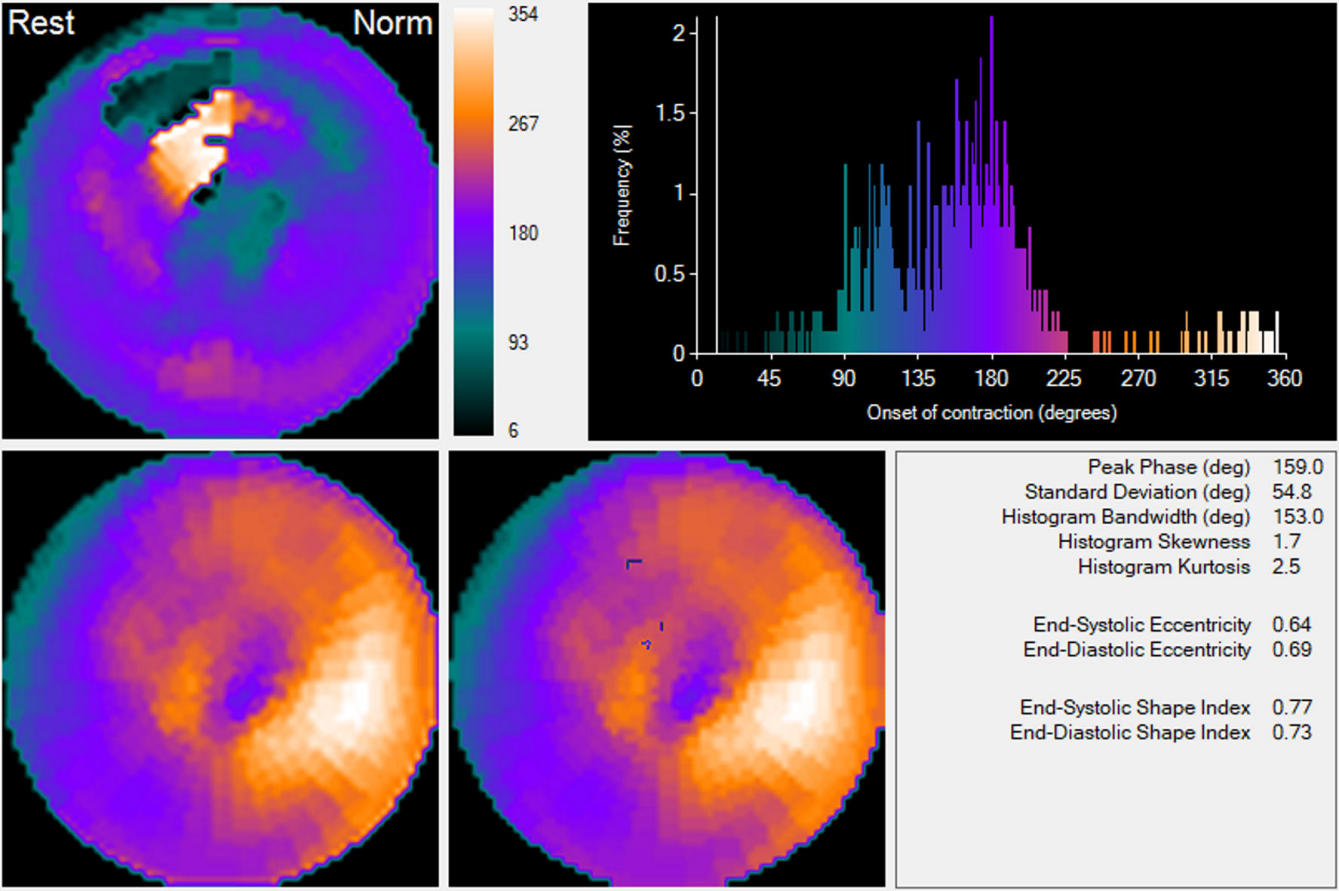
ECTb4 dyssynchrony results of a patient with LBBB and LVEF = 12% being evaluated for CRT (same as Fig. 2). Top left panel shows the phase polar map where the phase of each pixel, representative as the onset of mechanical contraction, is determined using the 1st Harmonic Fourier Transform and color-coded as described. Top right panel shows the phase histogram where the *x*-axis ranges from 0 to 360° normalized to the R-to-R time length for one cardiac beat. The *y*-axis is the number of pixels (frequency) with a specific onset of mechanical contraction. Note that the pixel color in the phase polar map is the same color shown in the histogram coinciding with the same onset of contraction. The wider the phase histogram the more dyssynchronous the ventricle. Bottom left polar map is the resting MPI map. Note the reduction in septal counts consistent with patients with severe LBBB. The bottom right polar map is the same MPI map except that the pixels that coincide with the white cursor in the phase histogram are blacked out. This is used to provide a dynamic display of the LV wave of mechanical contraction. Note that the cursor’s position is at approximately 190°, late in the global LV contraction. Also note the location of the blackened pixels in the inferolateral and inferior segments. The quantitative LVdys report shows the PSD to be 54.8°, > than the 43° recommended for CRT

The LV functional parameters used to identify potential responders to CRT were the LVEF (< 35% by echo) and the magnitude of intraventricular dyssynchrony (PSD > 43° by ECTb4^8^) at baseline before CRT. PSD is the standard deviation of the phase distribution (Figure 3), which was used as the preferred parameter to measure LV dyssynchrony. Note that even though the normal PSD cut-off with ECTb was measured as 24.4° for men and 22.2° for women^1^ it does not mean that patients that exceed this threshold are in heart failure or require CRT. Also, note that the PSDs measured by other algorithms are probably different from those by ECTb4.

The identification of the last contracting viable segment(s) by ECTb4 is used to identify the target region in the LV to place the CRT lead via venous access.^9^ The LV functional parameters, measured by ECTb4, used to identify improved clinical response 6 months post CRT were as follows^2^: an improvement in LVEF ≥ 5%, a reduction in ESV by ≥ 15% and or an improvement in LVdys SD by ≥ 4°.

LVEF and ESV were measured by ECTb4 in the conventional way. The amount of LVdys is measured as the PSD.^1^ Another parameter used to measure LVdys is the phase bandwidth, which is measured as the range in degrees which includes 95% of the elements in the phase histogram. The PSD parameter is preferred over the histogram bandwidth because it is less susceptible to phase noise. An example of a phase histogram from a typical patient being evaluated for CRT is shown in Figure 3.

**Figure 4:**
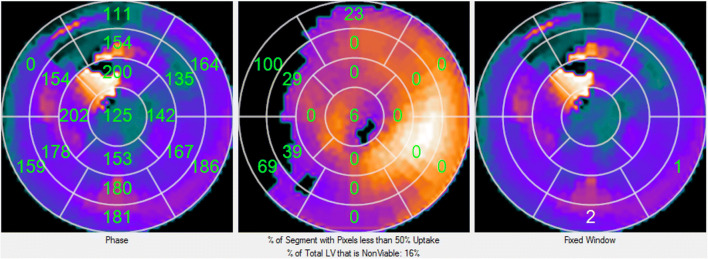
Automatic determination of the last viable segment(s) to contract (same patient as Figs. 2 and 3). This figure shows the polar maps used in the three-step process described. Left polar map is the phase polar map divided into the 17-segment model display showing the measured average phase for each segment. The middle polar map is the resting MPI distribution where each ≤ 50% of the maximum relative counts is identified as non-viable and the percent of non-viable pixels for each of the 17 segments is calculated. The right polar map is the phase polar where the last viable contracting segment is identified (with a 1) and another viable segments (2) within 10° of the last segment is also identified in case there is no practical venous access to the last viable contracting segment. Note that the visually seen late contracting segments in Fig. 3 (basal inferolateral and basal inferior) are the ones automatically determined by this phase guiding algorithm

The automatic determination of the last viable segment(s) to contract were determined as previously reported.^10^ Briefly, this is a three-step process (Figure 4). First, the phase polar map is divided into the 17-segment model and the average phase for each segment is determined representing the onset of mechanical contraction for that segment. Second, each pixel in the polar map of the resting perfusion distribution ≤ 50% of the maximum relative counts is identified as non-viable and the percent of non-viable pixels for each of the 17 segments is calculated as well as the percent of the total LV that is non-viable. Third, for each patient that the total LV that is non-viable is < 50%, and for only the non-apical segments that are in the anterior, anterolateral, inferolateral and inferior walls the algorithm determines the segment with the largest average phase and with the extent of viability > 50% as the last viable segment to contract. Other viable segments within 10° of the last segment are also identified in case there is no practical venous access to the last viable contracting segment.

## Conclusion

This report presents helpful tips used in the VISION-CRT trial intending to explain and guide the correct gMPI acquisition, processing and parametrization of synchrony-pertinent LV functional parameters that may assist in the identification of patients who respond to CRT and in the evaluation of the treatment. The procedures described in this report were used by the nuclear cardiology core laboratory at Emory University to generate the trial’s results and each of the individual trial sites independently followed this guidance to generate the site’s results. By following these procedures applied to the automated algorithms described here should ensure widespread use of reproducible dyssynchrony measurement techniques that overcome the disagreements reported in the PROSPECT trial when using echocardiographic techniques for the same purpose.^12^ The agreement between the Emory core lab results and those from the independent VISION-CRT sites is the subject of a future report.

## Electronic supplementary material

Below is the link to the electronic supplementary material.

Electronic supplementary material 1 (OPUS 373 kb)

Electronic supplementary material 2 (PDF 1219 kb)

## Abbreviations

CRT: Cardiac resynchronization therapy
ECTb4: Emory Cardiac Toolbox version 4
ES: End systole
ED: End diastole
LBBB: Left bundle branch block
LVdys: Left ventricular mechanical dyssynchrony
LVEF: Left ventricular ejection fraction
gMPI: Gated myocardial perfusion imaging
PSD: Phase standard deviation
