# Supplementary material for: Technical aspects of gated SPECT MPI assessment of left ventricular dyssynchrony used in the VISION-CRT study
Source: J Nucl Cardiol. 2020 May 11;28(3):1165–71. doi: 10.1007/s12350-020-02122-3 (PMC8249285; doi:10.1007/s12350-020-02122-3)
Supplement: Supplementary file 2 — Electronic supplementary material 2 (PDF 1219 kb) [file 12350_2020_2122_MOESM2_ESM.pdf]

Journal of Nuclear Cardiology | Official Journal of the American Society of Nuclear Cardiology

TECHNICAL ASPECTS OF GATED SPECT MPI ASSESSMENT OF LEFT VENTRICULAR DYSSYNCHRONY USED IN THE VISION-CRT STUDY

Amelia Jimenez-Heffernan MD, PhD, FEBNM, FASNC[a], Sadaf Butt MD[b], Claudio T Mesquita MD[c], Teresa Massardo MD[d], Amalia Peix MD, PhD, FACC, FASNC[e], Alka Kumar MD[f], Chetan Patel MD[g], Erick Alexanderson MD, FACC[h], Luz M Pabon MD[i], Ganesan Karthikeyan MD, MSc[g], Claudia Gutierrez, MD[j], Victor Marin MD[j], Ernest Garcia PhD[k] and Diana Paez MD, MSc[l].

[a]Hospital Universitario Juan Ramon Jimenez, Huelva, Spain; [b]Oncology and Radiotherapy Institute (NORI), Islamabad, Pakistan; [c]Hospital Universitario Antonio Pedro, Niteroi, Brazil; [d]Hospital Clinico Universidad de Chile, Santiago, Chile; [e]Nuclear Medicine Department, Institute of Cardiology, La Habana, Cuba; [f]Dr. B L Kapur Memorial Hospital, New Delhi, India; [g]All India Institute of Medical Sciences, New Delhi, India; [h]Instituto Nacional de Cardiologia Ignacio Chavez, Mexico DF, Mexico; [i]Fundacion Valle del Lili, Cali, Colombia; [j]Fundacion Cardioinfantil, Bogota, Colombia; [k] Emory University, Atlanta, GA; [l]Nuclear Medicine and Diagnostic Imaging Section, International Atomic Energy Agency, Vienna, Austria

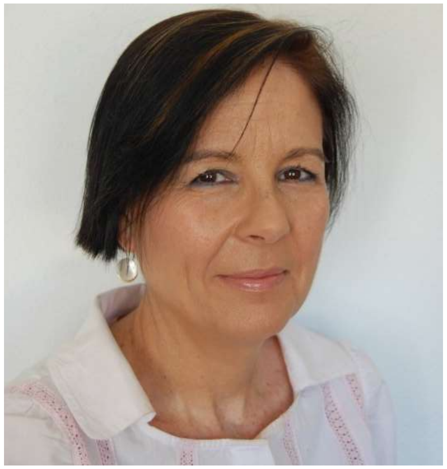

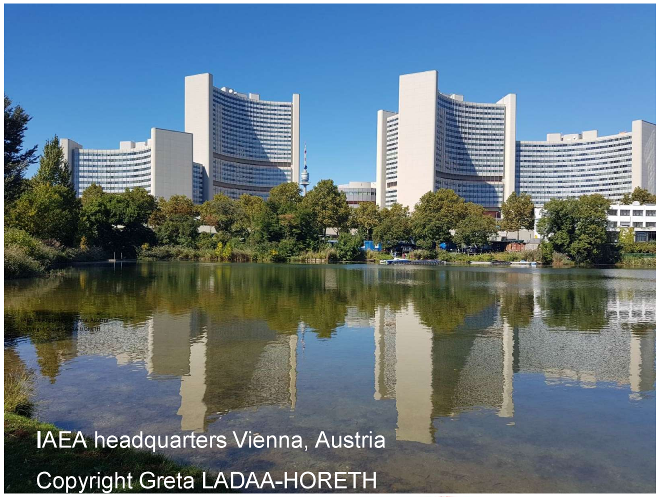

Copyright American Society of Nuclear Cardiology

Journal of Nuclear Cardiology | Official Journal of the American Society of Nuclear Cardiology

BACKGROUND

1. Describe the technical considerations undertaken in the IAEA VISION-CRT trial (identification of heart failure patients who will improve with cardiac resynchronization therapy) to assure valid results and the ECTb4 algorithms used to measure synchronicity-pertinent LV parameters.

2. Some of the descriptions are specific to ECTb4, but the quality control, the processing technical tips and algorithms descriptions are universal to technologists, physicians and industries interested in assessing LV mechanical dyssynchrony (LVdys).

3. Technical aspects of gated MPI acquisition, reconstruction & filtering, processing & quantification as well as processing quality control are reviewed.

Copyright American Society of Nuclear Cardiology

Journal of Nuclear Cardiology | Official Journal of the American Society of Nuclear Cardiology

Summary- 1

1. Acquisition: Both the accuracy of the ECG gating as well as the appropriateness

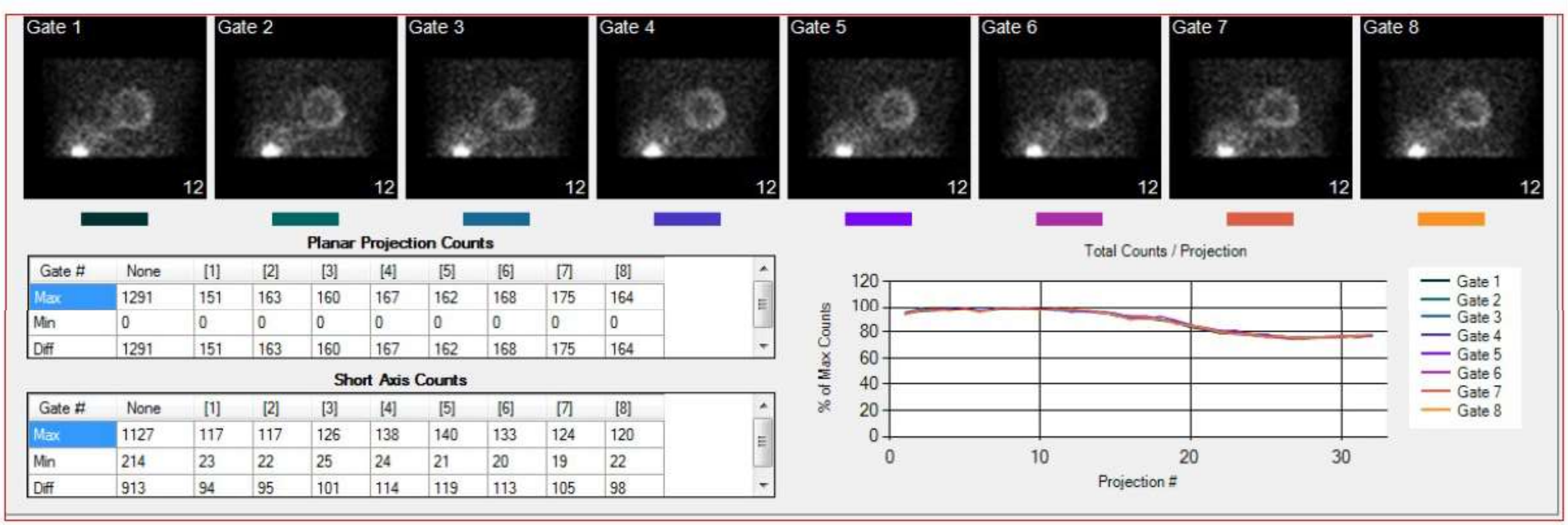

Quality control page in ECTb4 program provides information regarding gating and count statistics.

Technical Quality	Max Counts in Gated LV
**Adequate**	>80 (300 typical)
**Marginal**	20-80
**Inadequate**	<20

Copyright American Society of Nuclear Cardiology

Journal of Nuclear Cardiology | Official Journal of the American Society of Nuclear Cardiology

Summary- 2

1. Reconstruction & filtering: Standard OSEM reconstruction is used and the goal is to optimizing the number of counts per pixel. Additional spatial-temporal filtering is performed on the SA slices to reduce count noise.

2. gMPI Processing & quantification:

   The time-activity curve of a myocardial segment is equivalent to its temporal thickening curve.

   Both the measurements of thickening and onset of contraction measurements have been shown to be robust even in areas of hypoperfused myocardium allowing a good differentiation of patients with cardiac dysfunction from normal ones.

For Peer

Copyright American Society of Nuclear Cardiology

Journal of Nuclear Cardiology | Official Journal of the American Society of Nuclear Cardiology

Summary- 3

1. Processing quality control: Careful processing of gated SPECT MPI for dyssynchrony assessment is required to avoid and guarantee consistency. Therefore, a systematic approach is needed for optimal phase analysis

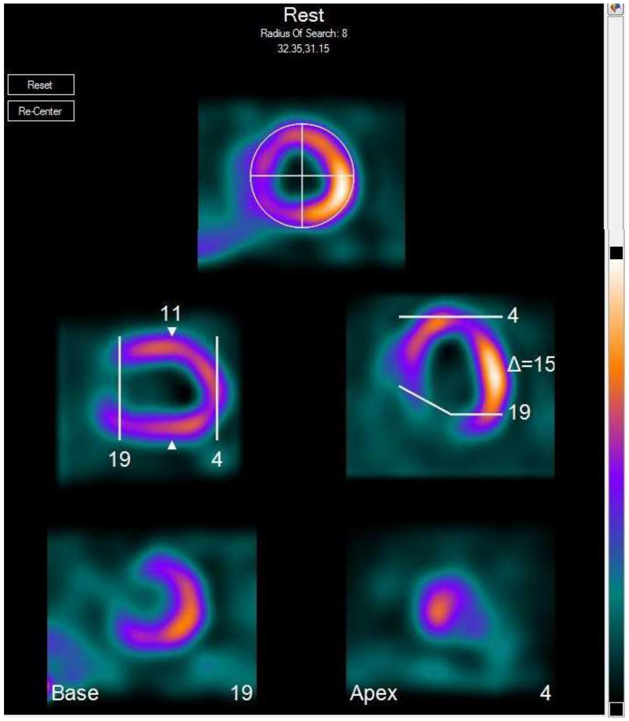

1) if the LVEF > 50% the base is allowed to move 2 slices in from ES to ED

2) if the LVEF > 35% and ≤ 50%, the base is allowed to move 1 slice from ES to ED

3) if the LVEF ≤ 35%, the base selection is not allowed to move

Correct base and apex determination to avoid phase artifacts

Copyright American Society of Nuclear Cardiology

Journal of Nuclear Cardiology | Official Journal of the American Society of Nuclear Cardiology

Summary- 4

1. Quantification to help guide and evaluate CRT:

   Phase SD is preferred over the histogram bandwidth because of its lower susceptibility to phase noise.

   The automatic identification of the last contracting viable segment(s) by ECTb4 is a 3-step process that is used to define the target region in the LV to place the CRT lead.

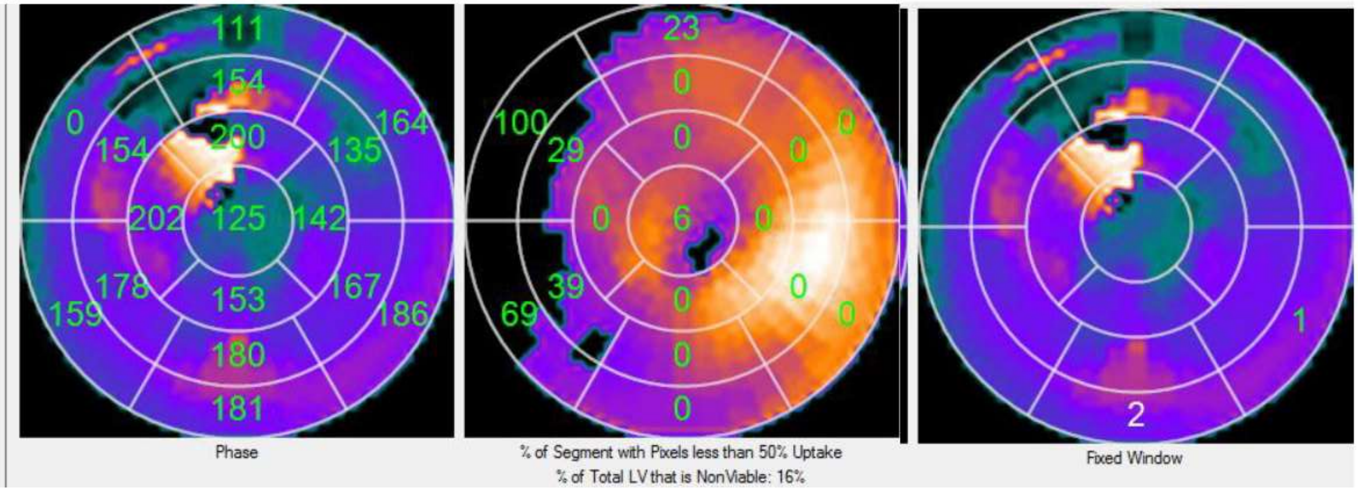

Automatic determination of the last viable segment(s) to contract

Copyright American Society of Nuclear Cardiology

Journal of Nuclear Cardiology | Official Journal of the American Society of Nuclear Cardiology

CONCLUSIONS & FUTURE DIRECTIONS

1. This report presents helpful tips used in the VISION-CRT trial intending to explain and guide the correct gMPI acquisition, processing and parametrization of synchrony-pertinent LV functional parameters that may assist in the identification of patients who respond to CRT and in the evaluation of the treatment.
2. The procedures described in this report were used by the nuclear cardiology core laboratory at Emory University to generate the trial's results and each of the individual trial sites independently followed this guidance to generate the site's results. By following these procedures applied to the automated algorithms described here should ensure widespread use of reproducible dyssynchrony measurement techniques that overcome the disagreements reported in the PROSPECT trial when using echocardiographic techniques for the same purpose[12].
3. The agreement between the Emory core lab results and those from the independent VISION-CRT sites is the subject of a future report.

Copyright American Society of Nuclear Cardiology
